# Novel diversity of Anaerolineae and Tepidiformia recovered from metagenomes of thermal microbial mats in Costa Rica

**DOI:** 10.3389/fmicb.2025.1693256

**Published:** 2025-12-11

**Authors:** Laura Brenes-Guillén, Daniela Vidaurre-Barahona, Kimberly Agüero, Andrés Ulloa, Yomara Zuñiga, Guillermo E. Alvarado, Lorena Uribe-Lorío

**Affiliations:** 1 Center for Research in Cellular and Molecular Biology (CIBCM), School of Biology, University of Costa Rica, San Jose, Costa Rica; 2 Center for Research in Cellular and Molecular Biology (CIBCM), University of Costa Rica, San José, Costa Rica; 3 School of Biological Sciences, Universidad Nacional, Heredia, Costa Rica; 4 Central American School of Geology, Research Center in Geological Sciences, University of Costa Rica, San José, Costa Rica; 5 Central American School of Geology, University of Costa Rica, San José, Costa Rica; 6 Laboratory of Atmospheric Chemistry Costa Rica, Universidad Nacional, Heredia, Costa Rica; 7 Center for Research in Cellular and Molecular Biology, School of Agronomy, University of Costa Rica, San José, Costa Rica

**Keywords:** candidate phyla, Chloroflexota, metagenomics, thermophiles, microdiversity

## Abstract

Tropical thermal and mineral springs are ideal for studying microbial life in extreme environments, their microbial diversity, and functional profiles. In this study, we investigated the abundance and genomic diversity of the phylum Chloroflexota in microbial mats from 33 thermal and acidic springs across Costa Rica using 16S rRNA gene amplicon sequencing and shotgun metagenomics. Our results demonstrated that pH and temperature are the main environmental drivers shaping the abundance and diversity of Chloroflexota. Acidic conditions favored the presence of Ktedonobacteria and the candidate division AD3, while thermal environments were dominated by unclassified Anaerolineae. From a subset of thermal springs, we reconstructed 72 metagenome-assembled genomes (MAGs), many of which represent previously uncharacterized lineages. Comparative genomic analyses revealed two novel families and seven new genera within Anaerolineae and a distinct lineage within Tepidiformia. We proposed the following names: *Ca.* Sittenfelaceae, *Ca.* Mariellaceae, and *Ca. Tepidiforma platanarica*. Functional annotation of Anaerolineae and Tepidiformia MAGs suggested a degree of functional redundancy. Genes associated with methanogenesis, dissimilatory nitrate reduction, sulfur metabolism, and methylotrophy were detected, while genes involved in photosynthesis, nitrogen fixation, and nitrification were absent. Unique gene clusters were identified in each family, and interestingly, 23% of these unique genes were of unknown function, highlighting the unexplored genetic potential of these organisms. Canonical correspondence analysis (CCA) revealed that temperature significantly influences the microdiversity of Anaerolineae. Despite their taxonomic novelty, these lineages exhibit strong functional redundancy across major metabolic pathways, where overlapping metabolic capabilities may confer stability under fluctuating conditions and support the persistence of diverse Chloroflexota populations. This study provides the first genomic dataset of Chloroflexota from Central American geothermal environments and highlights tropical geothermal springs as reservoirs of novel microbial diversity and functional potential.

## 1 Introduction

Thermal spring ecosystems share characteristics with early Earth conditions, and their distinct physical and chemical features promote the proliferation of particular microbial groups. These environments are an interesting source for exploring the impact of geographic isolation on prokaryote speciation and the distribution of microbial species that are adapted to high temperatures (Ward et al., 1998). Decades of biological research in these environments have focused on the taxonomy, phenotypic traits, and genetic characteristics of bacterial phyla such as Chloroflexota using culture-dependent techniques (Pierson and Castenholz, 1974; Giovannoni et al., 1987; Sekiguchi et al., 2003; Van Der Meer et al., 2010; Kochetkova et al., 2020; Spieck et al., 2020). However, our understanding of Chloroflexota’s abundance, taxonomy, and metabolism under thermophilic conditions has significantly advanced through metagenomic studies (Miller et al., 2009; Klatt et al., 2011; Xian et al., 2020; Bennett et al., 2022) and metatranscriptomic approaches (Liu et al., 2011; Klatt et al., 2013; Alcamán-Arias et al., 2018). These methods have provided valuable insights into the phylum’s metabolic potential, ecological niches, and previously unrecognized diversity. The abundance of Chloroflexota classes varies depending on the environment. For example, in certain thermal springs with temperatures above 64 °C, Roseiflexaceae and Chloroflexaceae can comprise up to 60% of the microbial community’s relative abundance (Uribe-Lorío et al., 2019). In contrast, in cold and acidic environments, such as acid mine drainage, some classes—such as AD3—may represent only 1–5% of the community (García-Moyano et al., 2015; Mesa et al., 2017), while others, such as the order Ktedonobacterales, can become predominant in silica-precipitating cave systems (Ghezzi et al., 2021). Despite these advances, current knowledge remains limited, particularly concerning tropical geothermal ecosystems.

Microbial mats are photosynthetic biofilms composed of stratified consortia of prokaryotes and microeukaryotes that develop at the sediment–water interface (Pan, 2021). Chloroflexota ranks among the most abundant photoheterotrophic phyla in microbial mats (Inskeep et al., 2013; Rozanov et al., 2017; Pagaling et al., 2012; Hamilton et al., 2019; Uribe-Lorío et al., 2019; Bennett et al., 2020; Xian et al., 2020). This phylum has been found to play a crucial role by (1) providing filamentous scaffolding that facilitates the development of biofilms under both aerobic and anaerobic conditions and (2) functioning as metabolically diverse microorganisms, including photoautotrophs, fermenters, organotrophs, obligate organohalide, and iron and nitrate reducers (Fincker et al., 2020). Consequently, it is involved in several metabolic pathways. The lifestyle diversity of Chloroflexota may confer competitive advantages over other bacteria, enabling it to withstand the strong selective pressure imposed by environmental conditions characterized by high ion concentrations and temperature.

The geotectonic context of Costa Rica is primarily controlled by the subduction of the Cocos plate beneath the Caribbean plate along the Middle America Trench and by the collision of the buoyant Cocos Ridge in the south (DeMets, 2001). This complex tectonic configuration produces an active volcanic arc that exhibits a high diversity of geothermal systems, with more than 200 thermal springs reported across the country, including acidic or silica-rich springs and bicarbonate- or sulfate-dominated springs (Alvarado and Vargas, 2017; Alvarado, 2021). Moreover, the complex tectonic and geographic setting of Costa Rica results in strong climatic and ecological gradients, producing remarkable microclimatic and landscape diversity even within individual mountain ranges (Alvarado, 2021; Alvarado et al., 2024). This geological variability in a tropical region is likely to shape the physicochemical niches that influence microbial diversity.

We hypothesize that environmental conditions are the main drivers influencing the structure and diversity of Chloroflexota communities in thermal and mineral environments and that some Chloroflexota lineages from thermal springs harbor distinct genetic reservoirs reflecting unique ecological adaptations and evolutionary divergence within the phylum. This study enhances our understanding of the structure and diversity of the Chloroflexota community in microbial mats from thermal and mineral springs in Costa Rica through shotgun metagenomic and 16S rRNA gene amplicon sequencing. To the best of our knowledge, this study represents the first genomic dataset of Chloroflexota from thermal springs in Central America.

## 2 Materials and methods

### 2.1 Site characteristics and sample collection

**Figure 1:**
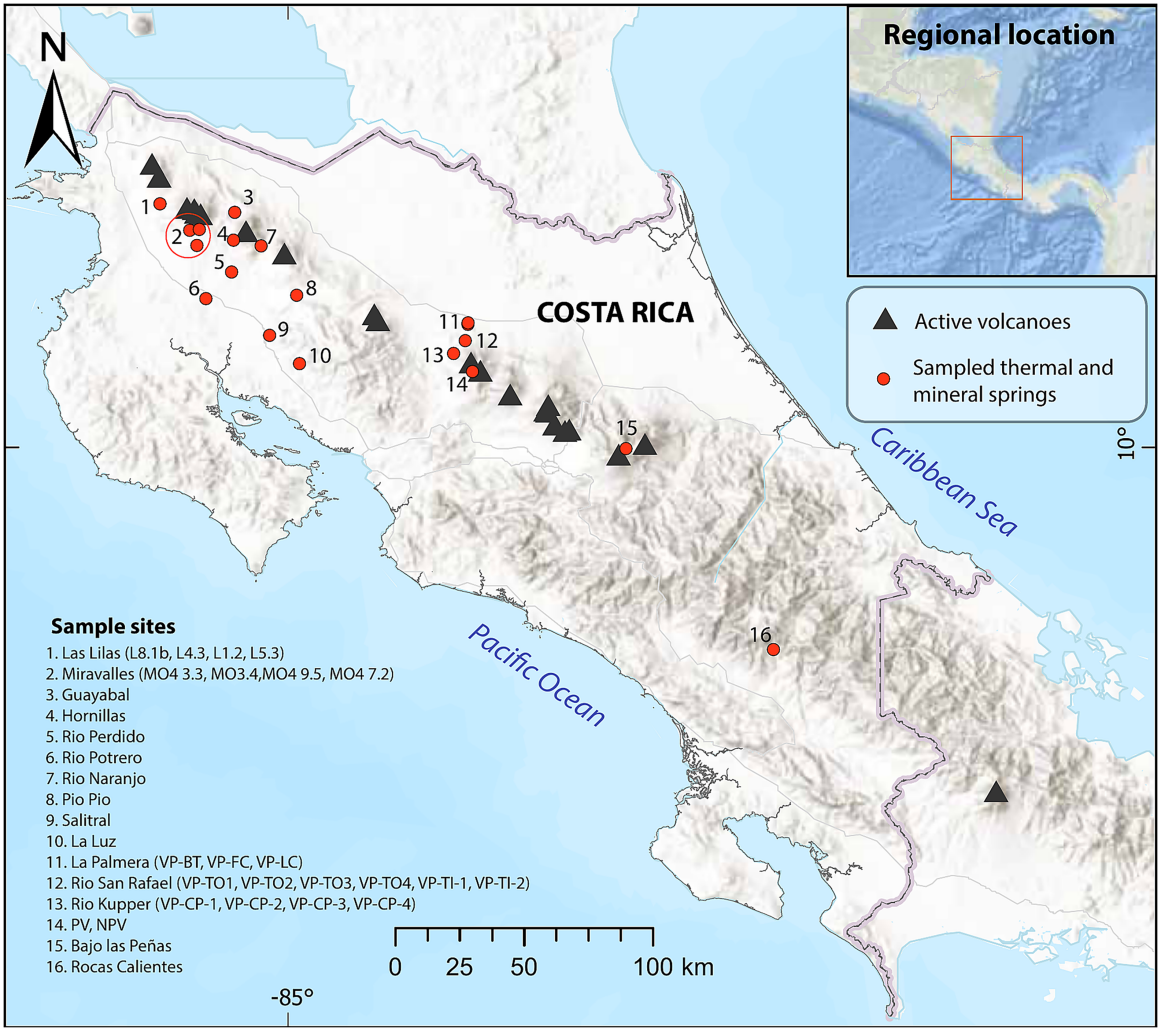
Geographic distribution of the sampled thermal and mineral springs (red dots) and active volcanoes (black triangles) in Costa Rica. The inset shows the regional location of Costa Rica in Central America.

We collected microbial mats with a surface area of 2–5 cm^2^ from 28 thermal and six mineral springs between 2004 and 2019 (Figure 1; Supplementary Table S1). The microbial mat samples were carefully scraped from submerged surfaces using sterile spatulas and transferred into sterile tubes. Temperature, pH, and conductivity were measured *in situ* using an Oakton multiparameter tester. Approximately 1 liter of water was collected in sterile plastic bottles and kept at 4 °C for chemical analyses. Chemical analyses of metal ions and sulfur were performed using Inductively Coupled Plasma Optical Emission Spectrometry. Flow injection analysis was used for nitrate (N-NO3) determination. The corresponding physicochemical parameters are detailed in Supplementary Table S1. The samples were preserved in tubes containing 50 mL of sucrose lysis buffer [0.75 M sucrose, 40 mM ethylenediaminetetraacetic acid (EDTA), pH 8, and 50-mM Tris–HCl, pH 8]. Initially, they were kept on melting ice and then transferred to the laboratory. Subsequently, they were preserved at −70 °C until DNA extraction.

### 2.2 Nucleic acid extraction

For DNA extraction, the samples were thawed on ice and agitated for 10 min using a DS LAB Orbital Shaker at 100 rpm. Subsequently, 1 g of material was processed with NucleoSpin Plant II (Macherey Nagel™) or DNeasy PowerSoil Pro Kit (Qiagen™), following the manufacturer’s instructions (Supplementary Table S1). DNA integrity was assessed by electrophoresis on 1.0% agarose gels and quantified using NanoDrop™ ND-1000. Then, the DNA extracts were stored at −20 °C.

### 2.3 Amplicon sequencing and data analysis

The DNA samples were sent to Macrogen Inc. in Seoul, South Korea, for the amplification of the 16S rRNA gene using the MiSeq Platform by Illumina Inc. The forward primer used was Bakt_341F: CCTACGGGNGGCWGCAG, and the reverse primer was Bakt_805R: GACTACHVGGGTATCTAATCC. These primers targeted the hypervariable V3–V4 regions (Mori et al., 2014), resulting in tags approximately 300 bp long. Raw sequence data obtained from the Illumina sequencing platform were processed using QIIME2 (version 2025.7) (Bolyen et al., 2019) and its plugins. In addition, the DADA2 plugin was used to denoise and dereplicate the paired-end sequences (Callahan et al., 2016) and to truncate reads (−p-trim-left 0, −p-trunc-len 280). The demux plugin was used for demultiplexing and viewing sequence quality. The uchime-ref method (reference-based chimera filtering with vsearch) was applied to identify chimeric feature sequences. The mitochondrial and chloroplast sequences were subsequently removed. Taxonomy was assigned to the amplicon sequence variants (ASVs) against the SILVA 16S/18S rRNA non-redundant reference dataset (SSURef 138.2 NR) (Quast et al., 2013) using the feature-classifier classify-learn method.

### 2.4 Statistical analysis of 16S RNA amplicon data

Multivariate analysis was performed using the Plymouth Routines in Multivariate Ecological Research (PRIMER7) (Clarke and Gorley, 2015). To evaluate differences in the structure of the Chloroflexota phylum across environmental gradients, we performed analysis of similarities (ANOSIM) based on the Bray–Curtis dissimilarity. Separate ANOSIM tests were conducted for pH and temperature ranges, and significance was assessed using 999 permutations. A shade plot was used for the visualization of the relationship between the sample groups and the 50 most relevant genera, as determined by PRIMER7. Hierarchical clustering was performed independently for both samples and genera. The samples were clustered based on the Bray–Curtis similarity index calculated from square root-transformed, standardized abundance data, while the genera were grouped using Whittaker’s index of association. The shading intensity in the plot represents the square root transformation of the absolute abundance of each genus in the respective samples.

### 2.5 Metagenomic sequencing and binning depuration

To study the functional and taxonomic diversity of prokaryotes across a range of temperatures, a subset of 14 metagenomes was sequenced from the thermal spring samples using the TruSeq Nano DNA Library Kit and HiSeq 4000 to obtain 100 bp paired-end reads. After sequencing, the raw reads were filtered using Trimmomatic (PE, SLIDINGWINDOW:4:20 MINLEN:100). Trimmed metagenomic reads were individually assembled using MEGAHIT v1.1.2 (min-contig-len 1000) (Li et al., 2015). Anvi’o interactive viewer v6.2 was used for metagenome binning (Eren et al., 2015). High-, medium-, and low-quality standards for metagenome-assembled genomes (MAGs) were assigned according to the criteria proposed by Bowers et al. (2017).

The completeness, contamination, and other metrics of MAGs were assessed using CheckM2 v1.0.1 (Chklovski et al., 2023). Only MAGs with completeness ≥ 50% and contamination < 10% were included. The taxonomy of the 72 MAGs was classified using GTDB-Tk v2.2.6 (Chaumeil et al., 2022) with GTDB r226 (Parks et al., 2022). We used BLAST (Altschul et al., 1990) to search the National Center for Biotechnology Information (NCBI) database to identify the sequence with the highest similarity for MAGs containing a 16S rRNA sequence (Supplementary Table S2). All sequences are available under BioProject PRJNA871231.

### 2.6 Comparative genomic analysis

Anvi’o interactive viewer v6.2 was used for phylogenomic and pangenomic analyses following the procedure described at http://merenlab.org/2016/11/08/pangenomics-v2/ and https://merenlab.org/2017/06/07/phylogenomics/ (Eren et al., 2020; Delmont et al., 2018), using the default parameters. For phylogenomic analysis, we used the default bacterial single-copy core gene collection, anvi’o Bacteria_71 (Lee, 2019). MUSCLE (Edgar, 2004) was used to generate multiple sequence alignments, and FastTree (Price et al., 2009; Version 2.1.10 SSE3) was used to infer approximately maximum-likelihood phylogenetic trees from the nucleotide alignments. Average nucleotide identity (ANI) between MAGs and reference genomes was calculated using PyANI (Eren et al., 2015). We conducted pangenomic analysis to identify conserved and unique genes in MAGs from thermal environments and reference genomes. The unique gene clusters found in each family based on the pangenomic analysis were annotated using eggNOG-mapper v2 (v.2.1.6) (Cantalapiedra et al., 2021) and the database COG224 (Galperin et al., 2025).

The phylogenetic tree was constructed using 16S rRNA sequences extracted from the MAGs and reference sequences obtained from GenBank, representing the closest phylogenetic groups of *Tepidiforma.* Evolutionary distances were calculated using maximum-likelihood inferences. Substitution models were compared using maximum-likelihood values (InL) in MEGA 6 (Tamura et al., 2013). Maximum-likelihood analysis was performed in MEGA 6, using a general time-reversible model with gamma-distributed and invariant site (G + I) rates, and bootstrap resampling was performed on 10,000 replicates. Predicted taxa for each MAG and BioSample, along with statistics, are provided in Supplementary Table S2. We used the Interactive Tree of Life (iTOL) v6 (Letunic and Bork, 2024) for tree visualization. To propose new families, genera, or species, we followed the requirements and recommendations for MAGs to serve as the nomenclatural type for species named under the SeqCode (Hedlund et al., 2022).

Given the functional analyses and the inherent limitations of working with MAGs rather than cultured bacterial genomes, we conducted a functional characterization on only MAGs classified as Anaerolineae and Tepidiformia. We used eggNOG-mapper v2 (v.2.1.6) for the functional annotation of MAGs (Cantalapiedra et al., 2021) with the database COG224 (Galperin et al., 2025). RStudio v2025.09.1 (Build 401) was used to plot functional profiles based on KEGG Orthology (KO) for nitrogen, carbon, and sulfur metabolism, as well as photosynthesis (Kanehisa et al., 2016). The list of KOs used in this analysis is provided in Supplementary Table S3. To characterize MAG microdiversity by analyzing metagenomic short-read alignments, we used InStrain v1.0.0 (Liao et al., 2023). The tool conducted microdiversity-aware genomic comparisons, specifically evaluating nucleotide diversity and breadth and identifying single-nucleotide variants (SNVs), including both synonymous and non-synonymous variants.

## 3 Results

### 3.1 Chloroflexota community profile in thermal and mineral microbial mats

**Figure 2:**
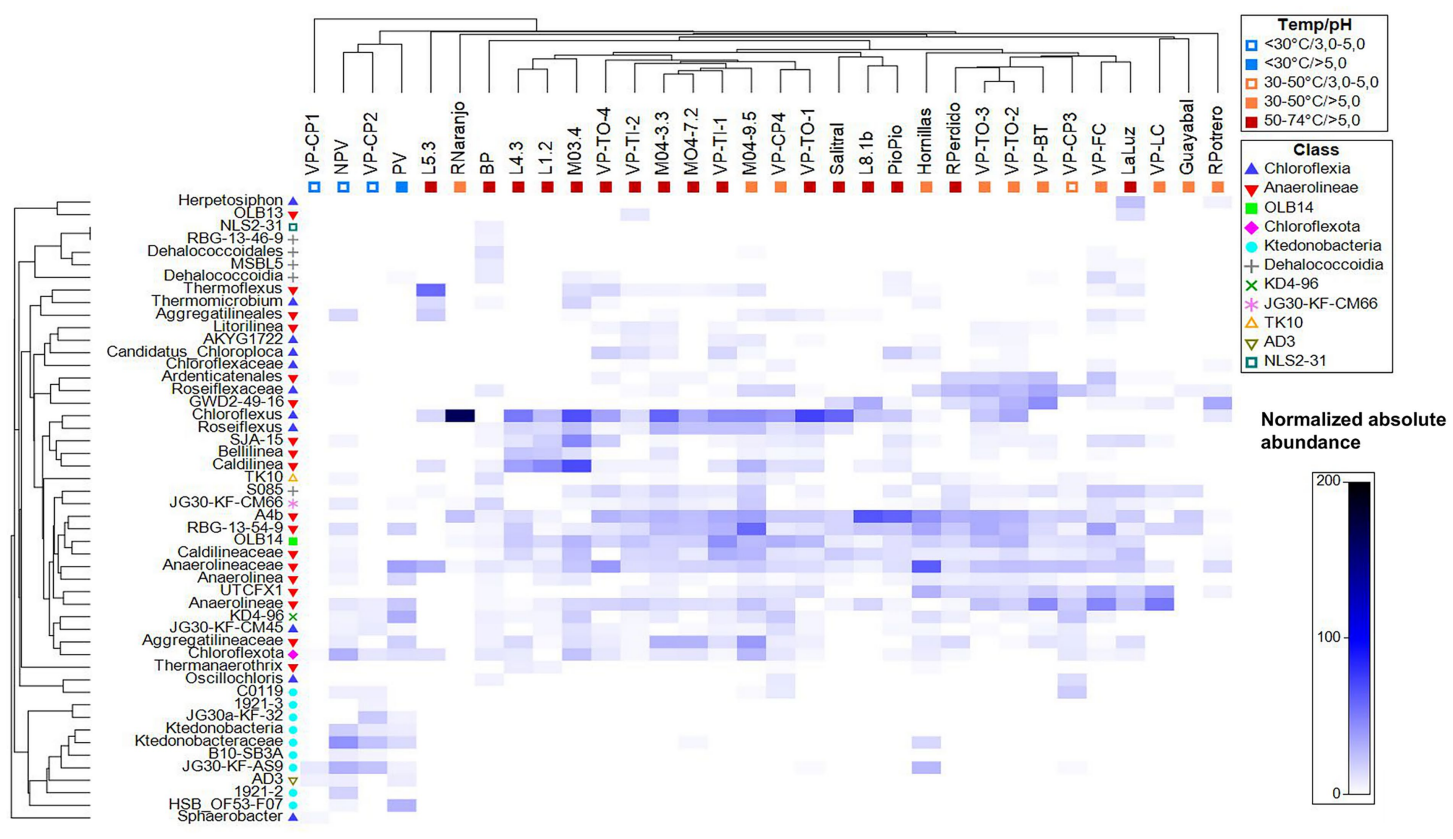
Shade plot (PRIMER v7) showing the 16S rRNA amplicon sequencing results (V3–V4) and the relationships between the sample groups and the 50 most abundant genera within the phylum *Chloroflexota* in microbial mats from mineral and thermal springs in Costa Rica. Shading intensities in the matrix indicate the square root-transformed absolute abundance of each taxon.

Chloroflexota comprised between 0.12 and 38% of the total prokaryotic community reads in both mineral and thermal springs (Supplementary Table S4). At the genus level, the main environmental drivers of Chloroflexota diversity in microbial mats were pH (*R* = 0.609; *p* = 0.00) and temperature (*R* = 0.38; *p* = 0.001). Members of the class Anaerolineae were predominant in microbial mats, followed by Chloroflexia and *Caldilinea* (Figure 2). Conversely, in cold and acidic environments (pH < 4), the distribution was Ktedonobacteria > Anaerolinae > Other taxa (Figure 2). The abundance of Ktedonobacteria, AD3, and unclassified Chloroflexota increased under acidic conditions. In thermal environments, the most abundant group was unclassified Anaerolineae, followed by uncultured lineages spanning all Chloroflexota classes, such as the Tepidiformia uncultivated lineage OLB14. These findings prompted the use of metagenomics to explore the taxonomy and functions of uncultivated Anaerolineae and Tepidiformia. At the ASV level, some ASVs of the genera *Chloroflexus* and *Caldilinea* were more abundant in Lilas samples (>74 °C), while ASVs of the same genera predominated in samples collected near Volcan Platanar (Supplementary Figure S1), suggesting that microdiversity patterns are driven by temperature and geographical location.

### 3.2 Comparative genomics of thermophilic Chloroflexota

We identified 72 MAGs associated with novel clades within the Chloroflexota phylum, based on relative evolutionary divergence, phylogenetic placement determined by GTDB-Tk, and a 95% ANI cutoff for species boundary (Supplementary Table S2). Furthermore, 86% of the MAGs had a completeness greater than 90% and a redundancy of less than 5% (high quality).

**Figure 3:**
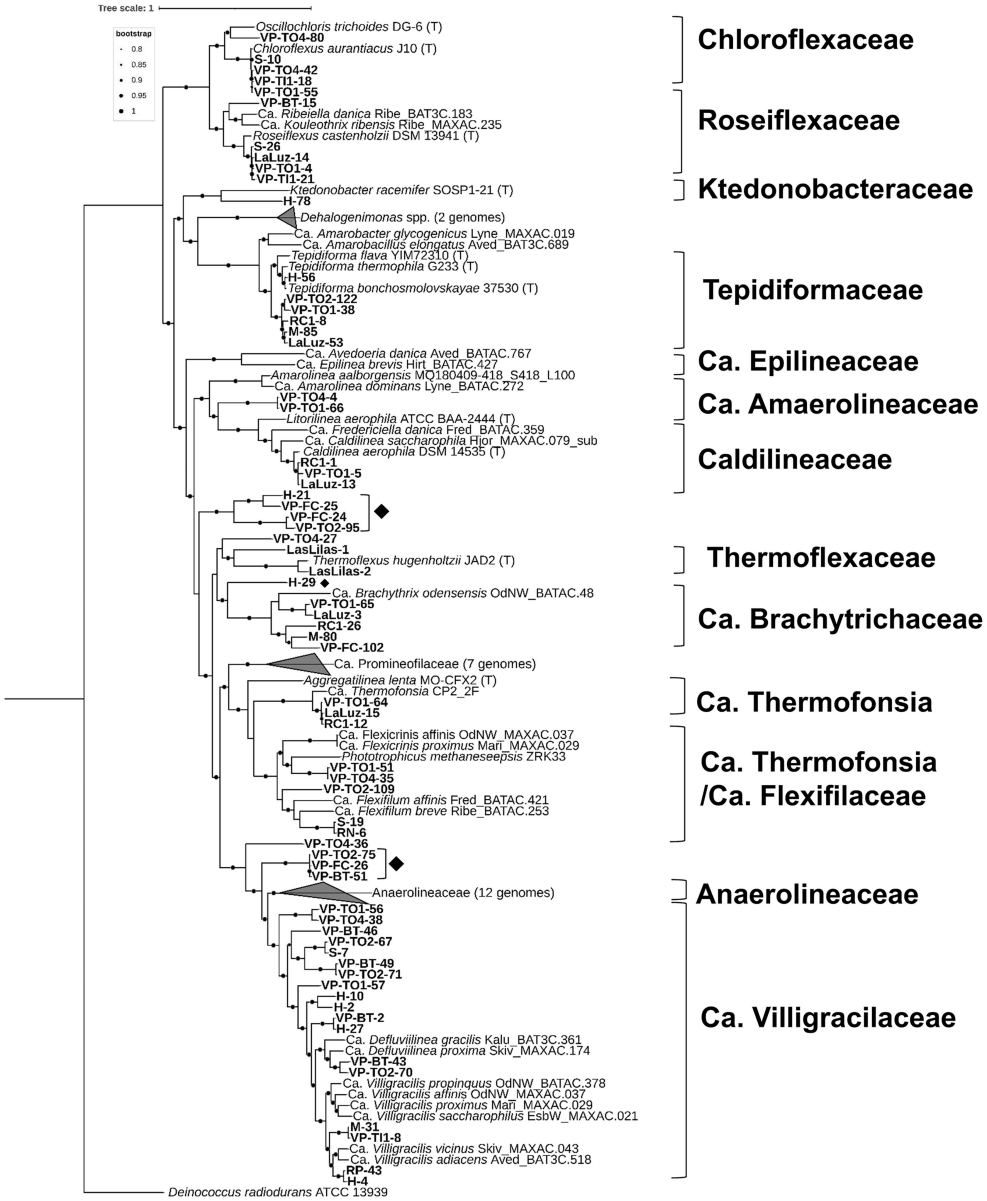
The maximum-likelihood phylogeny of Chloroflexota was constructed by concatenating a set of 71 single-copy core genes. Each taxon represents a distinct species-level group, with the highest-quality genome serving as a representative in cases where cultivated representatives are unavailable. *Deinococcus radiodurans* ATCC 13939 was included as the outgroup. Genomes derived from type strains are denoted with (T). The scale bar indicates the number of nucleotide changes per site, and supported branches are marked with dots at the nodes. All bootstrap values were greater than 0.80. Clusters with a rhombus correspond to MAGs that were classified only at the class level.

Anaerolineae is the class with the greatest unexplored taxonomic diversity. Of the 72 MAGs analyzed, 23 belonged to known and accepted thermophilic families, such as Chloroflexaceae (6), Roseiflexaceae (4), Thermoflexaceae (3), Caldilineaceae (3), Anaerolineaceae (1), and Tepidiformaceae (6). Phylogenomic analysis (Figure 3) revealed that 33 MAGs clustered within the candidate families found in wastewater treatment plants worldwide and deep-sea cold seeps (Petriglieri et al., 2023; Zheng et al., 2022). However, these MAGs represent new genera and species. We also identified two new clusters at the class level. This result may indicate the presence of novel families, genera, and species. GTDB-tk RED values were used to confirm the novelty of these taxonomic lineages (Figure 3; Supplementary Table S2).

### 3.3 Comparative genomics of the class Anaerolineae

**Figure 4:**
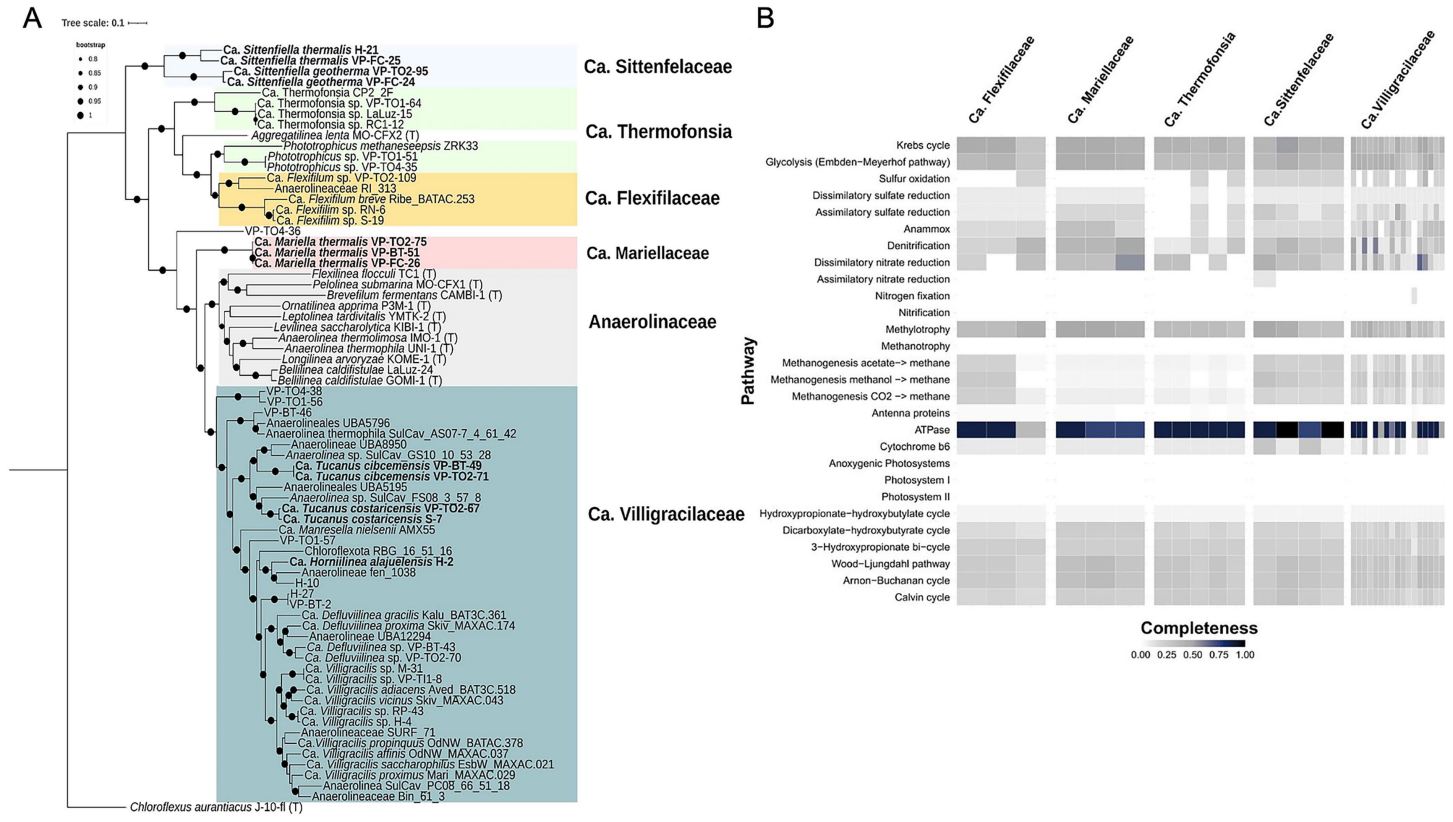
**(A)** The maximum-likelihood phylogeny of Anaerolineae was constructed by concatenating a set of 71 single-copy core genes. Each taxon represents a distinct species-level group, with the highest-quality genome serving as a representative in cases where cultivated representatives are unavailable. The color clusters correspond to the families. *Chloroflexus aurantiacus J-10-fl* was included as the outgroup. Genomes derived from type strains are denoted with (T). The scale bar indicates the number of nucleotide changes per site, and supported branches are marked with dots at the nodes. All bootstrap values were greater than 0.80. **(B)** The heatmap shows the completeness of each metabolic pathway for the 32 MAGs within each family. The colors indicate pathway-level completeness: black (complete), blue (75%), dark gray (50%), light gray (25%), and white (not present).

A total of 49 MAGs belonging to the class Anaerolineae were identified, according to GTDB-Tk classification and phylogenomic analysis. As mentioned, 49 MAGs were assigned to candidate families, such as *Ca.* Villigracilaceae (18 MAGs), Brachytrichaceae (five MAGs), *Ca.* Flexifilaceae (three MAGs), *Ca.* Thermofonsia (three MAGs), and *Ca.* Amaerolineaceae (two MAGs) (Figure 3). The remaining represented two proposed new candidate families within the Anaerolineae class. We proposed a new family, Candidatus Sittenfelaceae (Figure 4A). In addition, we also proposed two new genera and species within this family: *Ca. Sittenfiella geotherma* sp. nov. (type material: MAG VP-FC-24) and *Ca. Sittenfiella thermalis* sp. nov. (type material: MAG VP-FC-25). We also proposed the family *Ca.* Mariellaceae, and the genus and species *Ca. Mariella thermalis* (type material: MAG VP-TO2-75) (Figure 4A). The proposed new families were supported by at least three MAGs per group. MAGs belonging to these two new families were recovered from microbial mats near Volcan Platanar, with temperatures ranging between 37 °C and 50 °C.

Furthermore, four MAGs were classified within the genus *Ca. Villigracilis* and two within *Ca. Defluviilinea*, whereas the remaining 12 members of the *Ca. V*illigracilaceae family represented new genera. However, based on MAG completeness and redundancy, we only proposed three new genera and species. The first is *Ca. Tucanus cibcemensis* (type material: MAG VP-TO2-71), the second *Ca. Tucanus costaricensis* (type material: MAG S-7), and the third *Ca. Horniilinea alajuelensis* (type material: MAG H-2). All etymological descriptions can be found in the Supplementary data sheet S1. Genomes or MAGs recovered from sludge, deeply circulating subsurface aquifer fluids, and biofilms from sulfidic–thermophilic caves in Italy showed less than 70% average nucleotide identity (pyANI), with the Villigracilaceae new genera identified in our study (Figure 4A; Supplementary Table S5).

Despite the taxonomic divergence, almost all MAGs contained the complete set of genes encoding ATPase subunits but only partial sets of genes (25–50%) for glycolysis, the Krebs cycle, and the Calvin cycle. Our results highlight the presence of genes involved in methanogenesis, dissimilatory nitrate reduction, sulfur metabolism, and methylotrophy across all families (Figure 4B). However, MAGs did not have any genes involved in oxygenic or anoxygenic photosynthesis, nitrogen fixation, or nitrification. Genes involved in sulfur oxidation were not found in *Ca.* Mariellaceae. The gene *nifH* was detected in MAG VP-TO1-56, which belongs to a novel clade within the Villigracilaceae family. This clade was not assigned a genus-level taxonomic classification because none of the MAGs were more than 90% complete. In addition, genes encoding antenna proteins were found in all families except *Ca.* Sittenfelaceae (Figure 4B).

The pangenomic analysis revealed unique genetic features based on COG categories within each family (Supplementary Figure S2A). The *Ca.* Thermofonsia family had the largest unique genetic reservoir, with 908 unique genes assigned to COGs. This was followed by the *Ca.* Mariellaceae family, which contained 741 unique gene clusters (Supplementary Figure S2B). The analysis revealed that 23% of the unique genes detected in these MAGs were classified in category S (function unknown), 10% in category M (cell wall/membrane/envelope biogenesis), 9% in category T (signal transduction mechanisms), and 6% in category G (carbohydrate transport and metabolism). Notably, several putative hydrolases, including peptidases, putative regulatory proteins, and CRISPR-associated proteins, were identified.

### 3.4 Comparative genomics of thermophilic Tepidiformia

**Figure 5:**
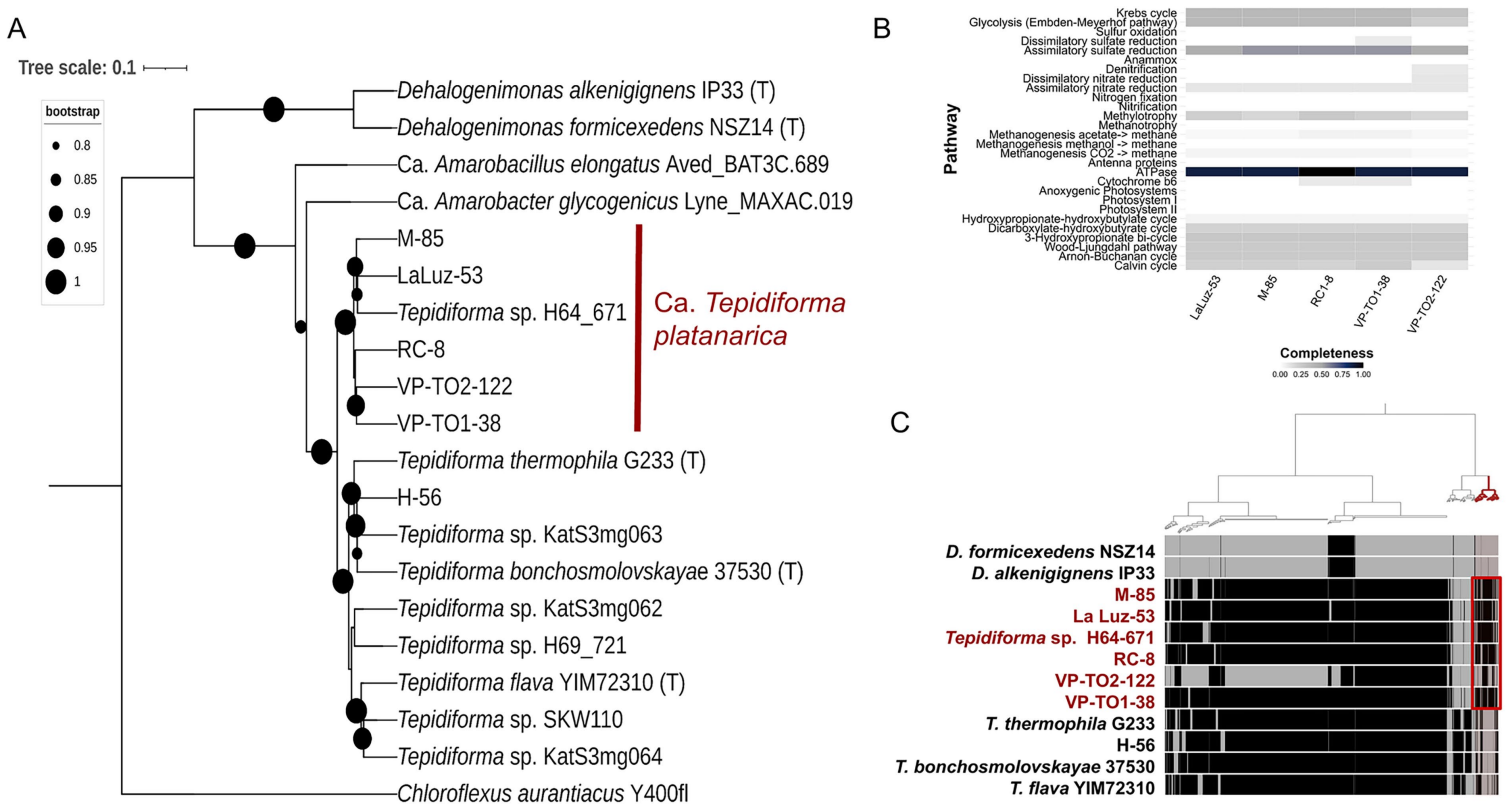
**(A)** The maximum-likelihood phylogeny of Tepidiformia MAGs, genomes, and *Dehalogenimonas* was constructed by concatenating a set of 71 single-copy core genes. *Chloroflexus aurantiacus* was included as the outgroup. Genomes derived from type strains are denoted with (T). The scale bar indicates the number of nucleotide changes per site, and supported branches are marked with dots at the nodes. All bootstrap values were above 0.80. **(B)** The heatmap shows the completeness of each metabolic pathway for the five *Ca. Tepidiforma platanarica* MAGs. The colors indicate pathway-level completeness: black (complete), blue (75%), dark gray (50%), light gray (25%), and white (not present). **(C)** Pangenomic analysis of MAGs and type strain genomes. The dendrogram was constructed based on the presence or absence of gene clusters. Each layer represents all genes (black) in a single genome. MAGs belonging to *Ca. Tepidiforma platanarica* are shown in red. The red color in the cladogram indicates unique gene clusters in *Ca. Tepidiforma platanarica.*

A total of six MAGs classified within the class Tepidiformia were grouped into two clades in the phylogenomic tree constructed for Tepidiformia MAGs, genomes, and *Dehalogenimonas* sequences (Figure 5A). The first clade included two type strains, *T. thermophila* G233 (Palmer et al., 2023) and *T. bonchosmolovskayae* 37530 (lineage OBL14, Kochetkova et al., 2020), along with MAG H-56, isolated from Hornillas. Based on ANI values, H-56 is 98% identical to *T. bonchosmolovskayae* 37530 and 92% identical to *T. thermophila* G233 (Supplementary Table S5). The second clade comprises MAGs recovered in this study and the *Tepidiforma* sequence H64_671. The H64_671 sequence was obtained from a metagenomic analysis of marine samples collected from the Nicoya Peninsula and the volcanic arc in northern and central Costa Rica. All these sequences had ANI values above 85% among themselves and with H64_671 (Wiegand et al., 2023) (Supplementary Table S6). However, none of the ANI values were above 95%, except between VP-TO2-122 and VP-TO1-38, whose identity was 99%. No associated MAGs were found in the *T. flava* YIM72310 clade. These results suggest that the second clade could be formed by a new species within the genus *Tepidiforma*, for which we propose the name *Candidatus Tepidiforma platanarica.* The phylogenetic tree based on 16S rRNA sequences supports these results (Supplementary Figure S3).

Similar to the functional profile of Anaerolineae, genes related to carbon, nitrogen, and sulfur metabolism were identified in the MAGs of *Candidatus Tepidiforma platanarica* (Figure 5B). In contrast to the Anaerolineae MAGs, no genes associated with the Anammox pathway were detected in *Ca. Tepidiforma platanarica*.

We conducted a pangenomic analysis to identify conserved and unique genes in *Tepidiforma* and *Dehalogenimonas* species. Our findings revealed a unique gene cluster shared by all MAGs of *Ca. Tepidiforma platanarica*, suggesting the uniqueness of these MAGs (Figure 5C). These unique genes were assigned to the categories of unknown function, coenzyme transport and metabolism, transcription, inorganic ion transport and metabolism, and amino acid transport and metabolism (Supplementary Figure S4). Notably, these include genes that encode hydrolases and CRISPR-associated proteins (Csd1 family). Furthermore, we identified one sigma factor and two anti-sigma factors that appear to be unique to *Ca. Tepidiforma platanarica*. Furthermore, the bacterial-type flagellum assembly gene *fliK* was found in three MAGs.

### 3.5 Temperature as a modulator of Anaerolineae microdiversity

**Figure 6:**
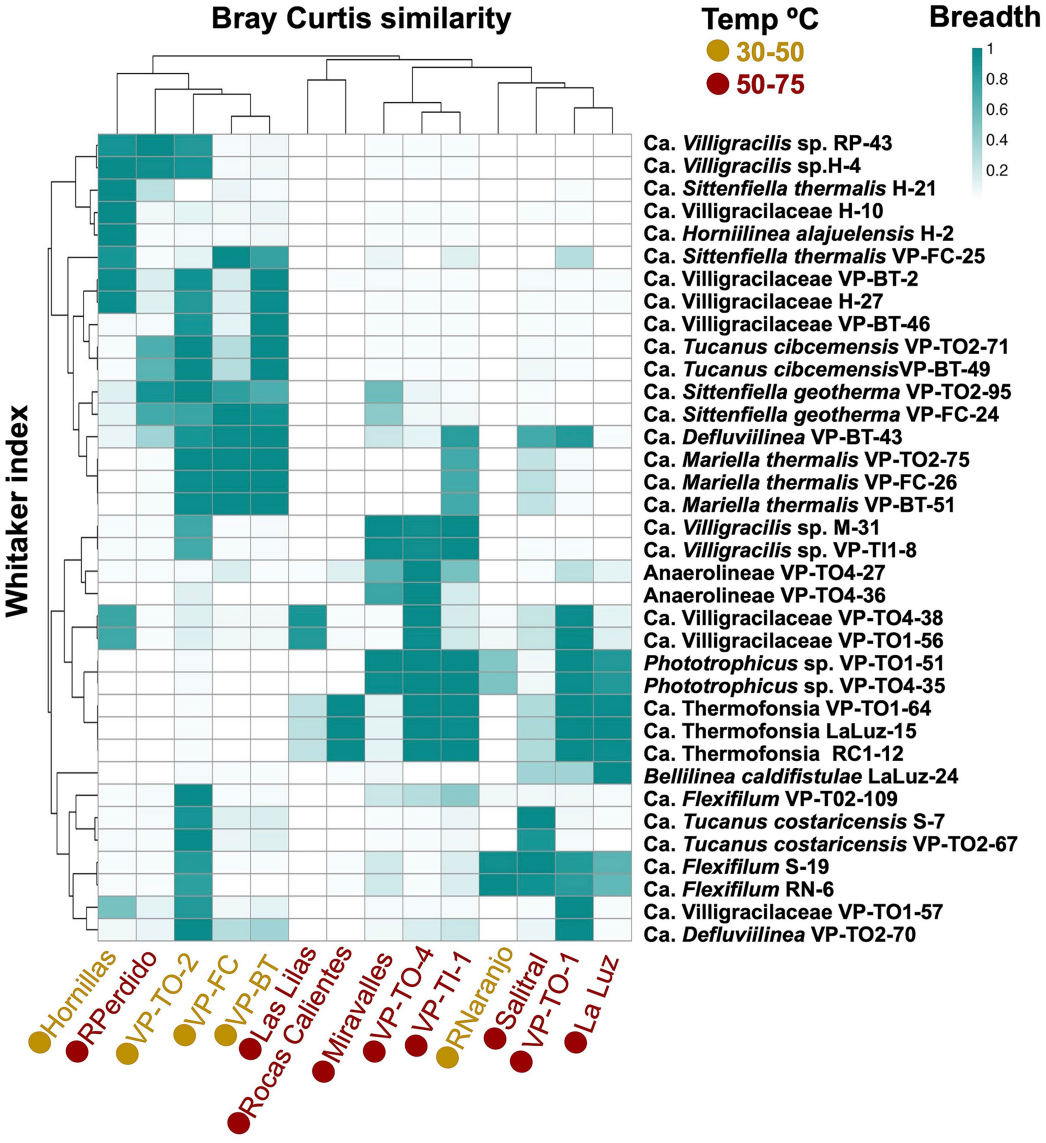
Breadth of MAGs in metagenomes from thermal springs in Costa Rica. The top cluster shows the grouping of the samples based on the Bray–Curtis similarity index, and the left cluster represents Whittaker’s index of association.

Canonical correspondence analysis (CCA) (Supplementary Figure S5) showed that temperature was a statistically significant factor influencing the distribution of Anaerolineae MAGs, explaining 52% of the variation in their abundance (*F* = 1.98, df = 1, *p* = 0.039). In contrast, ion concentration, pH, and conductivity were not significantly associated with changes in the abundance of MAGs in this class (*p* > 0.05). As shown in Figure 6, two distinct clusters of MAGs were identified: one group was associated with microbial mat samples at temperatures below 48 °C, while the other included MAGs that were more abundant at temperatures between 48 °C and 74 °C. Certain MAGs, such as *Bellilinea caldifistulae* LaLuz-24, *Ca.* Flexifilum VP-TO2-109, *Ca. Sittenfiella thermalis* H-21, and Anaerolineae VP-TO4-27, were exclusively detected in the metagenomes from which they were originally assembled, indicating potential habitat specialization (Figure 6).

## 4 Discussion

Chloroflexota is a widespread and abundant phylum commonly found in soils, biodigesters, sludge, wastewater, and freshwater ecosystems. The publication of numerous MAGs and 16S rRNA sequences may substantially expand the current phylogenetic tree of this phylum (Spieck et al., 2020; Xian et al., 2020; Bovio-Winkler et al., 2023; Petriglieri et al., 2023; Bovio-Winkler et al., 2024). These advances provide valuable tools for uncovering previously uncharacterized microbial lineages in microbial mats from thermal and mineral spring environments.

The findings of this study suggest that certain Chloroflexota lineages may possess the capacity to thrive in both acidic mineral and thermal environments. pH and temperature were identified as the primary drivers of prokaryotic community structure in mineral and thermal waters, as extensively documented in the literature (Tang et al., 2018; Uribe-Lorío et al., 2019; He et al., 2023). In our study, a large proportion of the variance remained unexplained (47%), suggesting that other abiotic or biotic factors also play a role in the abundance of Anaerolineae. Although no significant effects of sulfur, chloride, bromide, and fluoride concentrations on microbial assemblages were observed, water geochemistry could influence specific taxa and microdiversity within thermal communities (Barbosa et al., 2023; Debnath et al., 2023). Our analysis of amplicons showed that ASVs within the same genus exhibited differential abundance in geographically distant thermal springs (within 50 km). Similar results have shown that geographic location influences the diversity and distribution of protein-coding genes (Bennett et al., 2022).

Temperature may drive both genetic divergence at the finer tips of the phylogenetic tree (He et al., 2023) and functional specialization under different thermal conditions. These findings suggest that temperature is one of the primary drivers of Anaerolineae diversity and a key ecological force promoting microdiversification within this lineage. This is further supported by the particularly strong statistical separation between the lowest (30 °C) and highest (74 °C) temperature ranges. Comparative genomic results suggest that the diversity of thermophilic Anaerolineae and *Dehalococcoidia* MAGs may reflect evolutionary radiation within thermal niches, similar to patterns reported in other environments where these classes exhibit the strongest phylogenetic diversification within the phylum (Wiegand et al., 2023).

Pangenomic analysis showed that members of these clades contain unique genes with unknown functions. The high proportion of genes of unknown function within these novel MAGs underscores the vast, unexplored functional potential of thermophilic Chloroflexota. The universe of unknown sequences is remarkably diverse and more phylogenetically conserved than the known fraction, which is predominantly restricted to the species level (Vanni et al., 2022). This conservation may have facilitated the emergence and diversification of key microbial lineages (Méheust et al., 2022) and driven functional innovations (Rodríguez del Río et al., 2024). In fact, protein family presence/absence patterns broadly recapitulate the bacterial tree, suggesting that core sets of proteins, including those encoded by hypothetical proteins, have persisted since the divergence of lineages (Méheust et al., 2022). However, it has been suggested that rare genes exhibit low rates of positive selection at the species level, supporting a model in which most of the genetic variability observed within each protein family is selectively neutral (Coelho et al., 2022). Therefore, it is crucial to determine whether the unique accessory genes identified in thermophilic Chloroflexota lineages play a role in adaptation to high temperatures or in specific metabolic pathways.

Although Anaerolineae and Tepidiformia exhibit remarkable taxonomic diversity and contain unique genes in thermal springs, our analyses revealed a high degree of functional redundancy across the metabolic pathways examined. Similar decoupling between metabolic function and community taxonomic composition has been widely reported in microbial ecosystems (Tully et al., 2018; Louca et al., 2018; Tian et al., 2020). Functional redundancy is a common feature of microbial communities, associated with their ability to withstand environmental pressures, maintain temporal stability, and enable rapid recovery (Allison and Martiny, 2008, Qi et al., 2024, Rindi et al., 2025). In our study, we observed functional redundancy in the metabolism of carbon, nitrogen, methane, and sulfur. This redundancy is primarily a result of these pathways being essential for survival in environments with high ion concentrations, low nutrient availability, and elevated temperatures. They also exhibit versatility in the use of alternative electron acceptors (Vuillemin et al., 2024). This redundancy may also extend to other pathways, such as the degradation of complex organic compounds, which serve as important energy sources (Freches and Fradinho, 2024).

This study also contributes to databases by adding MAGs classified as Chloroflexota from thermal and mineral environments, including three novel families within Anaerolineae and Tepidiformia. However, there is a need to apply culturomics and enrichment approaches to characterize thermophilic bacteria in detail at the functional level and to expand the information from isolates. These approaches would also allow for the characterization of hypothetical proteins and unique gene clusters. Currently, they represent a significant portion of the genetic repertoire in these uncultivated taxa.

## Data Availability

The data presented in this study are publicly available. The data can be found here: https://www.ncbi.nlm.nih.gov/bioproject/PRJNA871231/, accession PRJNA871231.
