# Supplementary material for: Novel diversity of Anaerolineae and Tepidiformia recovered from metagenomes of thermal microbial mats in Costa Rica
Source: Front Microbiol. 2025 Dec 11;16:1693256. doi: 10.3389/fmicb.2025.1693256 (PMC12740240; doi:10.3389/fmicb.2025.1693256)
Supplement: Supplementary file 1 [file Data_Sheet_1.docx]

# Class Anaerolineae

## 1. Description of Candidatus Sittenfelaceae fam. nov.

**Etymology:** Sittenfelaceae (N.L. fem. pl. n.), named in honor of Dra. Ana Sittenfeld, Costa Rican microbiologist, for her contributions to the study of thermal environments in Costa Rica.

**Type genus**: *Candidatus Sittenfiella* gen. nov.

Members of this family are represented by metagenome-assembled genomes (MAGs) recovered from thermal environments in Costa Rica.

## 1.1 Description of *Candidatus Sittenfiella* gen. nov.

**Etymology:** *Sittenfiella* (N.L. fem. n.), named in honor of Dra. Ana Sittenfeld, for her contributions to microbial ecology of thermal environments in Costa Rica.

**Type species:** *Candidatus Sittenfiella thermalis* sp. nov.

### 1.1.1 Description of *Candidatus Sittenfiella thermalis* sp. nov.

**Etymology:** *thermalis* (L. fem. adj.), “of a thermal spring,” referring to the hot spring environment where the MAG were recovered.

**Type material:** MAG VP-FC-25, with an estimated completeness of 93.68% and contamination of 2.98%, containing 16S rRNA and 39 tRNAs.

### 1.1.2 Description of *Candidatus Sittenfiella geotherma* sp. nov.

**Etymology:** *geotherma* (N.L. fem. adj.), “geothermal,” referring to the geothermal origin of the sampling site.

**Type material:** MAG VP-FC-24, with an estimated completeness of 98.36% and contamination of 2.55%, containing 16S rRNA, 23S rRNA, and 46 tRNAs.

## 2. Description of Candidatus Mariellaceae fam. Nov.

**Etymology:** Mariellaceae (N.L. fem. pl. n.) (N.L. fem. pl. n.), named in honor of Dra. Marielos Mora López, Costa Rican microbiologist, for her contributions to the study of thermal environments in Costa Rica.

**Type genus**: *Candidatus Mariella* gen. nov.

Members of this family are represented by metagenome-assembled genomes (MAGs) recovered from thermal environments in Costa Rica.

### 2.1 Description of *Candidatus Mariella* gen. nov.

**Etymology:** named in honor of Dra. Marielos Mora López, for her contributions to microbial ecology of thermal environments in Costa Rica.

**Type species:** *Candidatus Mariella thermalis* sp. nov.

2.1.1 Description of *Candidatus Mariella thermalis* sp. nov.

**Etymology:** *thermalis* (L. fem. adj.), “of a thermal spring,” referring to the hot spring environment where the MAG were recovered.

**Type material:** MAG VP-TO2-75 with an estimated completeness of 92% and a contamination of 1.44 % and contains 16S rRNA and 35 tRNAs.

## 3. Family Candidatus Villigracilaceae (proposed by [Petriglieri](https://journals.asm.org/doi/10.1128/msystems.00667-23#con1) *et al.,* 2023)

## 3.1 Description of *Candidatus Tucanus* gen. nov.

**Etymology:** Tucanus (N.L. masc. n.), named after the Tucano river, Costa Rica, where the microbial mat from which the MAGs were recovered was found.

**Type species:** *Candidatus Tucanus costarricensis* sp. nov.

Members of this genus are represented by metagenome-assembled genomes (MAGs) recovered from thermal environments in Costa Rica.

3.1.1 Description of *Candidatus Tucanus costarricensis* sp. nov.

**Etymology:** *costarricensis* (L. adj.), referring to Costa Rica, the country where the MAGs where recovered.

**Type material:** MAG S-7 with an estimated completeness of 99.83% and a contamination of 5 % and contains 16S rRNA and 48 tRNAs.

3.1.2 Description of *Candidatus Tucanus cibcemensis* sp. nov.

**Etymology:** *cibcemensis* (L. adj.), refers to the CIBCM (Center for Research in Cellular and Molecular Biology) at the University of Costa Rica, the research center where the investigation was conducted.

**Type material:** MAG VP-TO2-71 with an estimated completeness of 99.86% and a contamination of 1.56 % and contains 36 tRNAs.

3.2 Description of *Candidatus Horniilinea* gen. nov.

**Etymology:** *Horniilinea* (N.L. fem. pl. n.), named after the Hornillas, the place where the microbial mat from which the MAGs were recovered was found.

**Type species:** *Candidatus Horniilinea costarricensis* sp. nov.

Members of this genus are represented by metagenome-assembled genomes (MAGs) recovered from thermal environments in Costa Rica.

3.2.1 Description *of Candidatus Horniilinea alajuelensis* sp. nov.

**Etymology:** *alajuelensis* (L. adj.), refers to the Costa Rican province of Alajuela, where the MAGs were recovered.

**Type material:** MAG H-2 with an estimated completeness of 100% and a contamination of 0.05 % and contains 47 tRNAs.

# Class Tepidiformia

## Genus Tepidiforma (proposed by Kochetkova *et al.,* 2020).

1.Description of *Candidatus Tepidiforma platanarica* sp.nov.

**Etymology:** *platanarica* (L. adj.), referring to the Platanar Volcano, a volcano near the thermal spring where the MAGs were recovered.

**Type material:** MAG VP-TO1-38 with an estimated completeness of 99.17% and a contamination of 3.27 % containing 16S rRNA, 23S rRNA, and 51 tRNAs.

Members of this species are represented by metagenome-assembled genomes (MAGs) recovered from thermal environments in Costa Rica.
