# Supplementary material for: Novel diversity of Anaerolineae and Tepidiformia recovered from metagenomes of thermal microbial mats in Costa Rica
Source: Front Microbiol. 2025 Dec 11;16:1693256. doi: 10.3389/fmicb.2025.1693256 (PMC12740240; doi:10.3389/fmicb.2025.1693256)
Supplement: Supplementary file 2 [file Supplementary_file_1.docx]

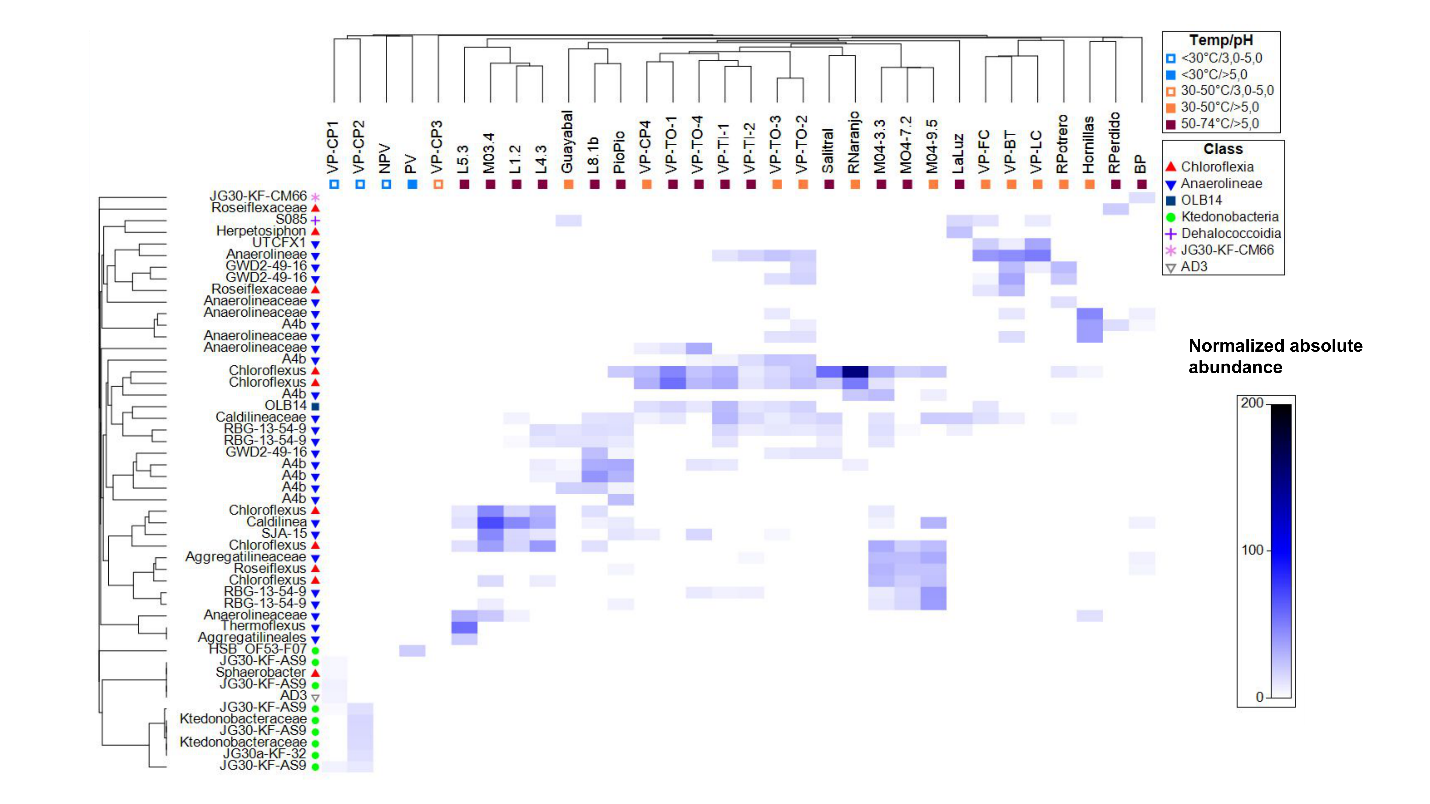


Figure S1. Shade plot (PRIMER v7) showing the results of 16S rRNA amplicon sequencing (V3–V4) and the relationships between sample groups and the 50 most abundant ASVs within the phylum *Chloroflexota* in microbial mats from mineral and thermal springs in Costa Rica. Shading intensities in the matrix indicate the square root transformed absolute abundance of each taxon.


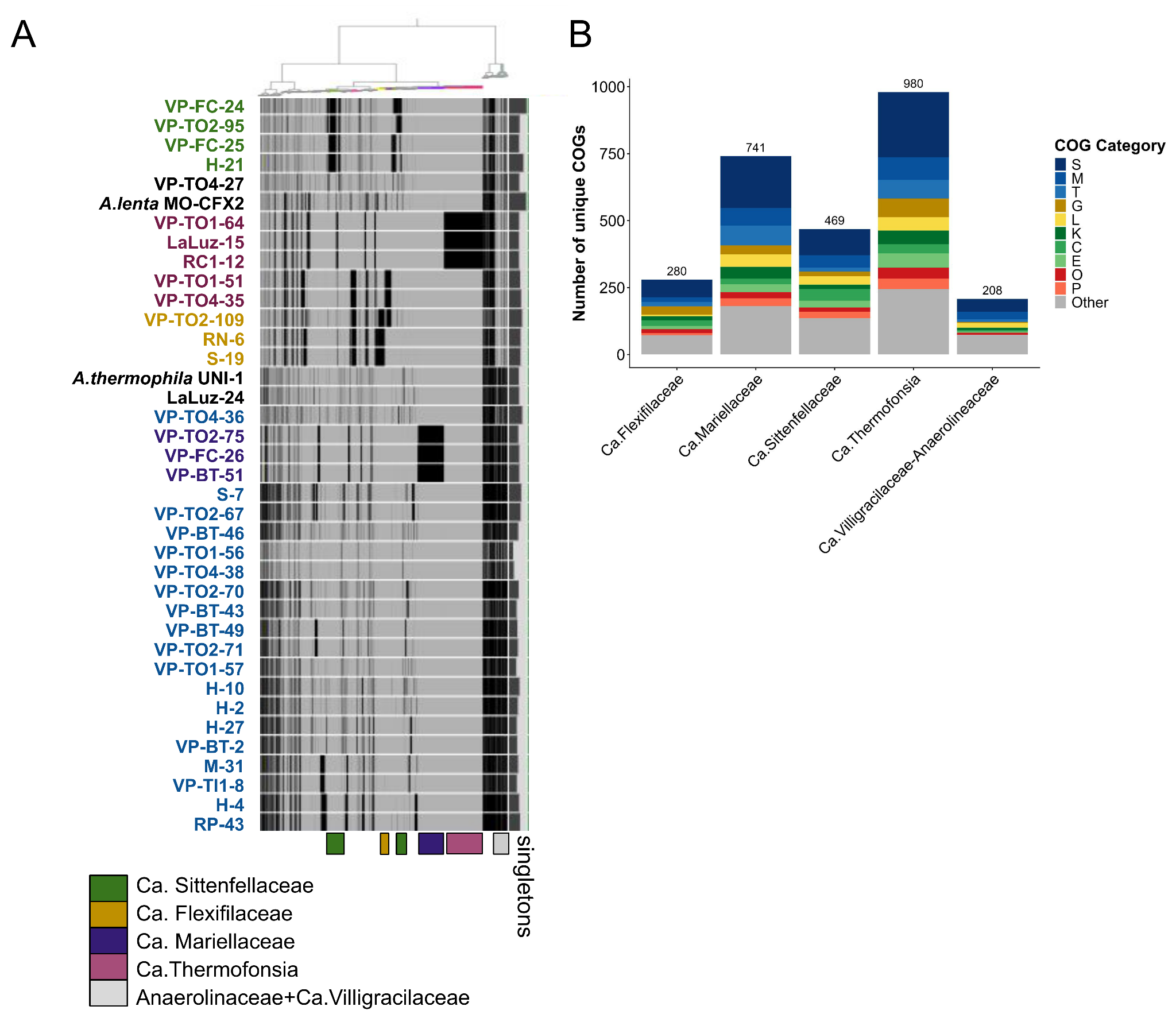


Figure S2. A. Pangenomic analysis between MAGs and *Anaerolinea thermophila* UNI-1 and *Aggregatilinea lenta* MO-CFX2 genomes.The dendrogram was constructed based on the presence or absence of gene clusters. Each layer represents all genes (black) in a single genome. The colors represented gene clusters that were considered unique by family. B. Number of unique gene clusters in each family and the Cluster of Orthologous Groups of proteins (COG) classification.


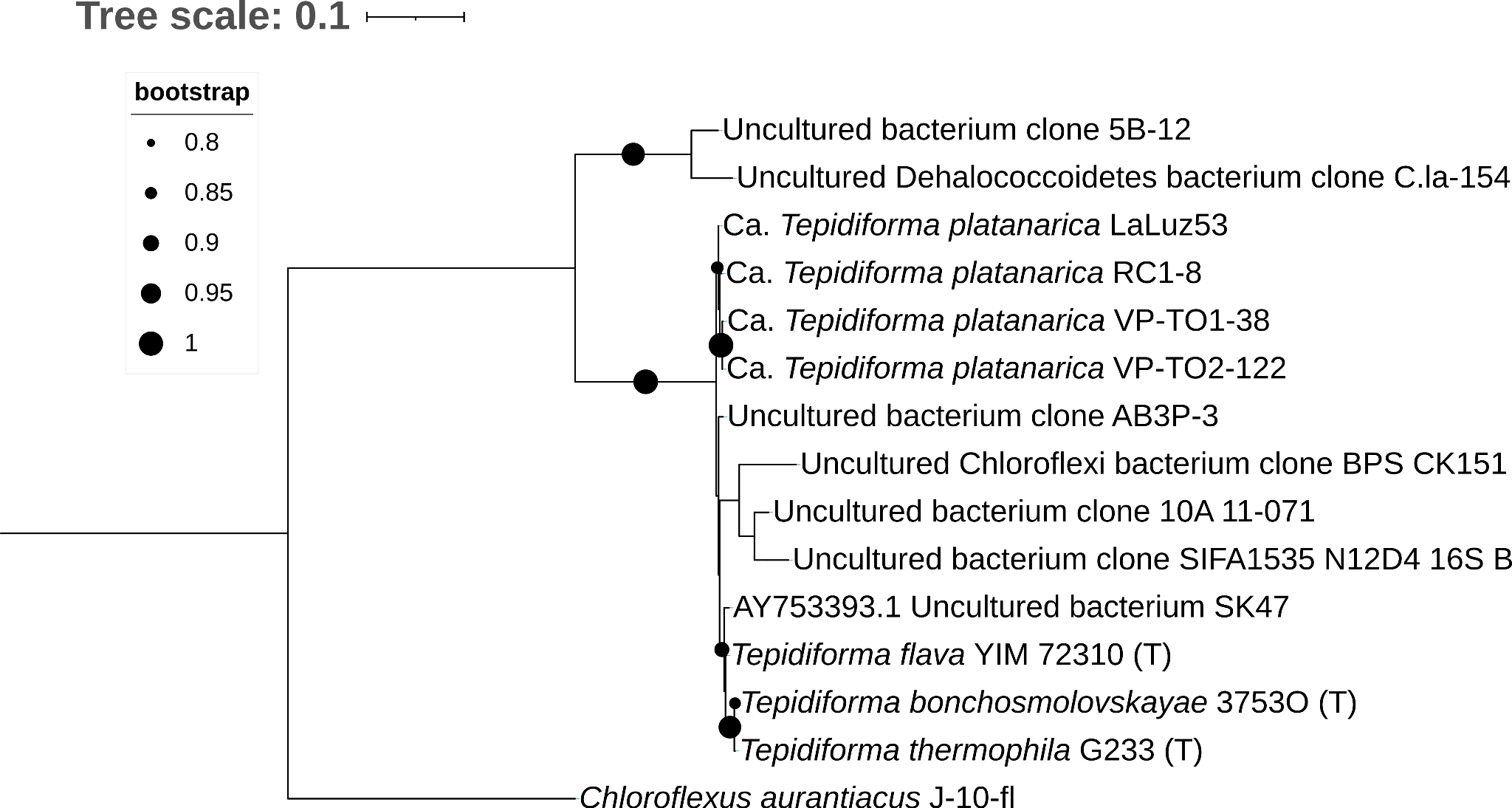


Figure S3. Maximum Likelihood tree based on 16S rRNA gene sequences showing the positions of Ca. *Tepidiforma platanarica*. *Chloroflexus aurantiacus* J-10-fl was included as an outgroup.. Bar shows 0.05 substitutions per nucleotide.


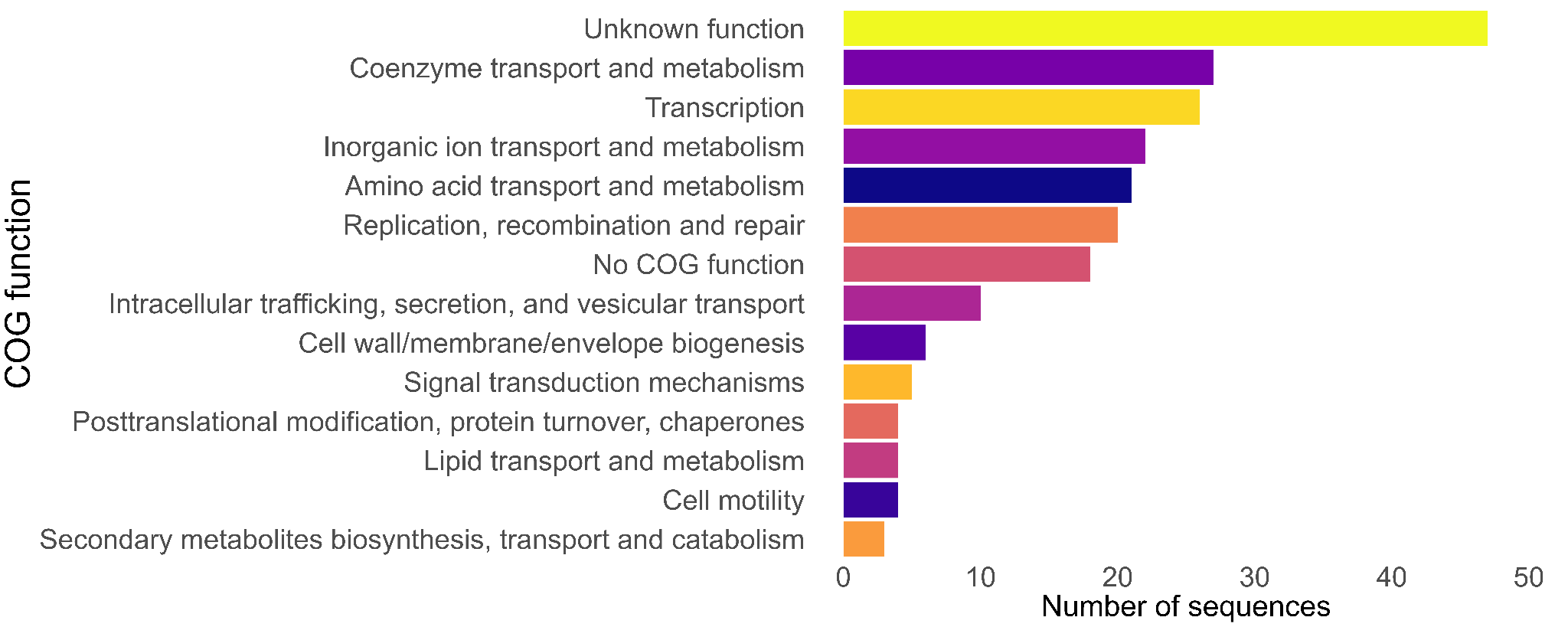


Figure S4. Number of unique gene clusters in Ca. *Tepidiforma platanarica* and the Cluster of Orthologous Groups of proteins (COG) classification.


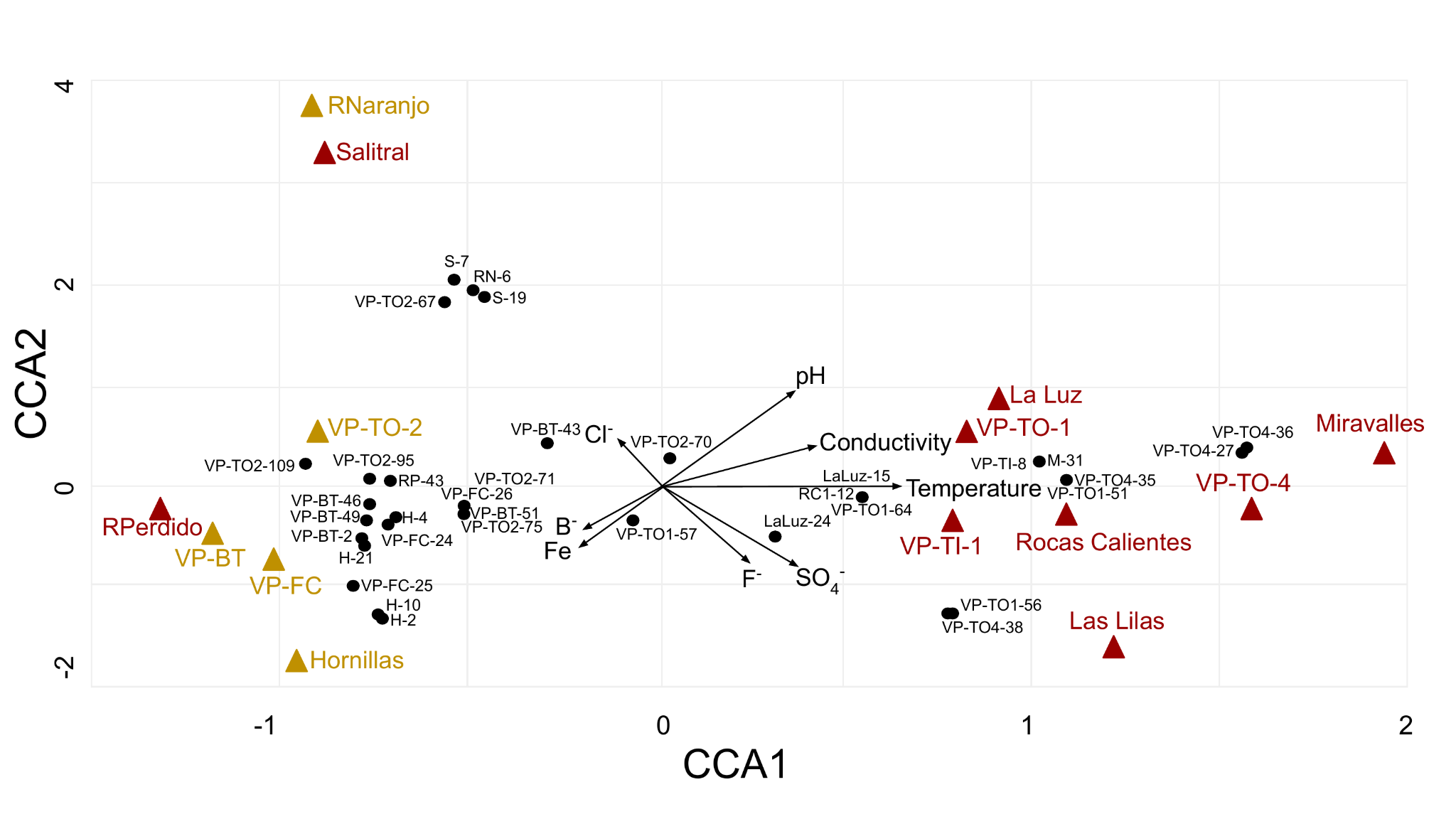


Figure S5. Canonical Correspondence Analysis (CCA) plot shows the relationship between the breadth of Anaerolineae MAGs and environmental variables. The variables are represented by black arrows. The MAGs are shown in blue, the sample identifiers in red, and the black dots represent the MAGs' points. Temperature was the only statistically significant factor influencing the distribution of Anaerolineae MAGs, explaining 52% of the variation in their abundance (F = 1.98, df = 1, p = 0.039). The red triangles indicate temperatures between 50 °C and 74 °C, while the brown ones indicate temperatures between 30 °C and 50 °C.
